# Poor Quality of Life in HIV-Infected Men Who Have Sex with Men is Associated with Excess-Type Constitution of Traditional Chinese Medicine

**DOI:** 10.1155/2023/9445111

**Published:** 2023-01-16

**Authors:** Li-Lan Liao, Chien-Chun Wang, Chien-Lung Wu, Yi-Shun Chu, Yi-Chang Chou, Chung-Hua Hsu

**Affiliations:** ^1^Institute of Traditional Medicine, School of Medicine, National Yang Ming Chiao Tung University, Taipei, Taiwan; ^2^Department of Chinese Medicine, Taipei City Hospital, Linsen Chinese Medicine and Kunming Branch, Taipei, Taiwan; ^3^Department of Infection, Taipei City Hospital, Linsen Chinese Medicine and Kunming Branch, Taipei, Taiwan; ^4^Department of Education and Research, Taipei City Hospital, Taipei, Taiwan; ^5^Institute of Public Health, National Yang Ming Chiao Tung University, Taipei, Taiwan

## Abstract

**Introduction:**

This study explored the pathological constitution as it relates to low quality of life in HIV-infected MSM patients, as a reference for clinical treatment.

**Methods:**

It had a cross-sectional research design using structured questionnaires to collect data, including patient's basic data, CD4^+^, CD4^+^/CD8^+^ ratio, Wang Qi constitution, and WHOQOL-BREF-Taiwan version questionnaires. We considered the association between constitutions and quality of life of HIV-infected MSM patients. *Results and Discussion*. The project accepted 203 HIV-infected MSM participants. The three most common pathological constitutions were Yang deficiency 15.5%, yin deficiency 13.1%, and qi deficiency 11.2%. The study determined scores for various quality of life domains: psychological (13.44 ± 2.27), social relationship (13.81 ± 2.80), physiological (14.43 ± 2.41), and environmental (14.78 ± 2.21). The TCM constitution is strongly correlated with the quality of life. Excess constitution had the worst quality of life. Comparing the infected time over one year with the time of <0–2 weeks, the adjusted odds ratios (AOR) were determined for abnormal CD4^+^ and CD4^+^/CD8^+^ ratio (OR: odds ratio: 0.03, 0.07, respectively, *p* < 0.001). Compared with the Gentleness constitution, there is a significant difference between the Deficiency and Excess constitution in sleep status and negative mood by multiple regression analysis (*p* < 0.001).

**Conclusion:**

The Excess constitutions was correlated with worse quality of life. Even if the immune system was restored, the psychosocial domain, sleep status, and negative mood were not improved.

## 1. Introduction

Quality of life (QOL) can be defined as a person's satisfaction and subjective well-being living in what she or he thinks is the most important area [1]. QOL is an important outcome indicator of HIV treatment. Compared with the general population, HIV-infected patients have lower quality of life due to life-long medications, suffering from side effects and anxiety/depression [2, 3]. A meta-analysis of 9,000 people living with HIV/AIDS (PLWHA) found that nearly 58% of HIV-infected persons have a high degree of sleep disturbance, and those with sleep disturbance also show more depression and health problems [4]. PLWHA in Taiwan have worse QOL in terms of physical, psychological, and social relationships than the general population (all *p* < 0.001) [5]. On average, published research works indicate that 60% of HIV-infected patients use complementary and alternative medicine (CAM) to treat health problems related to HIV infection [6]. Of these, MSM and HIV-infected patients with severe infection symptoms and longer duration of illness more commonly use CAM to prevent or alleviate HIV-related symptoms, reduce treatment side effects, and improve quality of life [7–11].

Chinese herbal medicine (CHM) is a type of CAM widely used in Taiwan's medical system. Relevant studies have shown that higher cumulative doses taken by HIV-infected/highly active antiretroviral therapy (HAART) patients increase the risk of hyperlipidemia, cardiovascular disease, respiratory disease, and diabetes [12]. In addition, during HAART treatment, patients who took CHM had a lower incidence of hyperlipidemia and cardiovascular disease than those who did not take CHM [13]. Studies have also shown that traditional Chinese medicine (TCM) can alleviate signs and symptoms related to HIV-infected symptoms, improve quality of life, extend long-term survival, alleviate adverse side effects of antiviral drugs, promote immune reconstitution, and improve laboratory results [14, 15].

Constitution is the core value of TCM, and it depends on the result of the innate and acquired environment by integrating the characteristics of physiological functions and mental states. Wang Qi classified the TCM constitution into nine constitutions, namely, Yang deficiency (Yang), Yin deficiency (Yin), Qi deficiency (Qi), Phlegm-dampness (Pw), Dampness-heat (Dh), Blood stasis (Bs), Special diathesis (Sp), Qi-stagnation (Qs), Gentleness (G). These were derived from classification standards extracted from complex constitutional phenomena. Constitutional identification shows the direction of treatment and provides core techniques for assessing health conditions and supports research on the relationship between constitution and disease [16–19]. Previous studies used Wang Qi's TCM constitution questionnaire to explore the correlation between cardiovascular disease, hypertension, diabetes and other diseases and specific TCM Constitution, and the association between moderate and severe cancer fatigue and TCM Constitution. The constitution identity can be used as an appropriate choice for the basis of TCM to prevent and treat diseases [20, 21].

Although the quality of life of HIV-infected patients is lower than that of people in general [3], there is no research discussing the relationship between TCM constitution and quality of life in HIV-infected patients. This study explores the correlation between the distribution of HIV-infected MSM patients in TCM constitution and quality of life in Taiwan, and what type of constitution is a risk factor for lower score quality of life, to develop a reference for treatment and preventive strategies.

## 2. Materials and Methods

### 2.1. Study Participants

The recruiting period was from January 2019 to December 2019. We invited all people diagnosed with HIV infected through MSM in the outpatient Department of Infection, Taipei City United Hospital, Linsen Chinese Medicine and Kunming Branch, to participate in this study. Patients must fulfill all the following criteria to be eligible for the study: those who are diagnosed with HIV (Centers for Disease Control and Prevention, revision of AIDS in 1993), those aged over 20 years (inclusive), those with clear cognition, and those who are able and willing to complete the study and provide written informed consent. The study design was reviewed and approved by the Institution of Taipei City Hospital Research Ethics Committee (IRB number: TCHIRB-10707110) and received certification by the clinical trials by the government (clinical trial number: NCT05299775). The Wang Qi's nine kinds of TCM constitution and WHOQOL-BREF-Taiwan version (World Health Organization Quality of Life, Brief Taiwan version) Questionnaires were authorized by Wang Qi and Yao Kaiping, respectively.

### 2.2. Data Collection

The recruited cases used the last 2 weeks as a reference point to judge their subjective quality of life and constitution related issues. The following are information from the hospital's medical records database: years, education, occupation, marital status, time of diagnosis, route of HIV infection, and CD4^+^, CD4^+^/CD8^+^ ratio. The inspection time for CD4^+^ and CD4^+^/CD8^+^ ratio was within 2 weeks before and after the recruited time. CD4^+^ < 292 cells/*µ*L, CD4^+^/CD8^+^ ratio < 0.6 was regarded as an abnormal value (Beckman Coulter).

### 2.3. Study Assignment of Questionnaires

#### 2.3.1. The WHOQOL-BREF-Taiwan Version is Widely Used to Measure Quality of Life

It includes physical health aspects (Ph, including original physiological and independent; items 3, 4, 10, 15, 16, 17, and 18), and psychological aspects (Ps, including psychological/spiritual/religious/personal beliefs; items 5, 6, 7, 11, 19, and 26), social relationship aspects (So, items 20, 21, 22, and 27^*∗*^), and environment aspects (En, items 8, 9, 12, 13, 14, 23, 24, 25, and 28^∗^). There are 28 items in the questionnaire, with 2 being local questions for Taiwan. The local questions of the society and environment are indicated by ^∗^.

Each item uses a five-point scale to score. The scores range from 1 (worst condition) to 5 (best condition), except for 3 items (Ph1, Ph2, and Ps6), which have reverse scores. Two scales (0–100 and 4–20, with a higher score indicating better QOL) can be transformed into domain scores for each domain. We used the 4–20 scale in this study, the higher scores indicating better quality of life. The reliability (0.70 to 0.77) and validity (0.53 to 0.78) of the questionnaire reached statistically significant levels (*p* < 0.01) [23]. These 4 domains, common sleep status (Ph5), and negative mood (Ps6), were used as clinical symptoms in this discussion.

#### 2.3.2. TCM Constitution Questionnaire by Wang Qi

It includes 9 basic types of constitution, Yang deficiency (7 items), Yin deficiency (8 items), Qi deficiency (8 items), Phlegm dampness (8 items), Damp-heat (6 items), Blood stasis (7 items), Special diathesis (7 items), Qi depression (7 items), and Gentleness constitution (8 items). Except for the Gentleness constitution, all others are considered pathological constitutions. One question is judged by Likert five-point scale from 1 point (none) to 5 (always). Nine kinds of TCM constitution—the constitution type must be judged according to the standards: original score = sum of the scores of items for each constitution, and then the original score is converted into a conversion score [conversion score = ((original score number of answer items)/answer items × 4) × 100].

Judgment of constitutions: Gentleness is a normal constitution and others are pathological constitutions. Gentleness constitution conversion score ≥60 points and other pathological constitution conversion score <30 points is considered Gentleness constitution; pathological constitution conversion score ≥40 is considered pathological constitution, the higher the conversion score, the more obvious the constitution trend, if any for more than 2 pathological constitutions, the highest score is used to judge the constitution [17, 23].

This study used these nine constitutions to explore the distribution of constitutions in these subjects. In addition, Yang deficiency, Yin deficiency, and Qi deficiency were combined into the Deficient Constitution, and phlegm dampness, damp-heat, and blood stasis were combined into the Excess Constitution.

### 2.4. Statistical Analysis

Demographic and other data were collected and analyzed using IBM SPSS Statistics for Windows, version 24.0 (IBM Crop.Armonk, NY). Pearson's chi-square test and Fisher's exact test were used to detect significant differences between years, infected time, CD4^+^, CD4^+^/CD8^+^ ratio, and different constitution distribution. For all parameters considered in the study, the approximation to normal of the distribution of the population was tested by the Shapiro–Wilk test and statistics for kurtosis and symmetry. As results were asymmetrically distributed, nonparametric tests were used. Data were expressed as median and interquartile range (IQR). The Kruskal–Wallis test followed by Dunn's post hoc test was used to detect significant differences between years, infected time, and quality of life.

The Mann–Whitney *U* Test was used to detect significant differences between CD4^+^, CD4^+^/CD8^+^ ratio, and quality of life. Logistic regression used parameters to discuss the odds ratio between CD4^+^, CD4^+^/CD8^+^ ratios by years, education, occupation, TCM constitution, and infection time. Multiple linear regression determined correlations between independent variable years, education, occupation, TCM constitution, and infected time as related to the sleep status and negative mood.

## 3. Results

The relevant parameters were as follows: The total recruited cases was 232, the number of valid cases was 203, and invalid was 26 (including those with incomplete personal basic information, biochemical data and questionnaires). So, the dropout rate was 12.5%.

### 3.1. Demography and Baseline Characteristics

There are 203 participants. Average age is 32.5 years, with a range from 20 to 49 years. The period of illness is divided into 3 stages (according to the date of diagnosis), ≤2 weeks, >2 weeks to one year, and more than one year. 73.9% of patients has been diagnosed for more than 1 year, 46% is 30–39 years, 61.5% has college education or above, 12.8% is unemployed, 5.4% and 23.6% has abnormal CD4^+^ or CD4^+^/CD8^+^ ratio, respectively, for more than one year of infection (see Table 1).

### 3.2. TCM Constitution Distribution

In the overall distribution of the TCM constitution, Gentleness accounted for 21.7%, and the pathological constitution was mainly composed of Yang deficiency, Yin deficiency, and Qi deficiency, accounting for 15.8%, 13.3%, and, 11.3%, respectively. The Excess constitution of Qi depression and phlegm dampness were the most common, accounting for 9.4% and 8.9%, respectively.

In Table 2, the authors divide the constitution into Deficiency, Excess, Special diathesis, and Gentleness constitution. 40.4% has Deficiency constitution, 27.6% has Excess constitution, 10.3% has Special diathesis, and 21.7% has Gentleness constitution. There is no significant relationship between the distributions of years, rank, infection time, and constitution (Fisher's exact test, *p*=0.63 and 0.18). There is also no significant relationship between CD4^+^, CD4^+^/CD8^+^ ratios and the distribution of constitutions (ANOVA, *p*=0.62 and *p*=0.58).

### 3.3. Quality of Life Score

Table 3 shows that the overall HIV-infected MSM quality of life has an average score of 14.09 ± 2.37, 13.13 ± 2.83, 13.69 ± 2.94, and 14.26 ± 2.19 in the physical, psychological, social, and environmental domains. The psychological domain has the lowest score. There are no significant differences between years and infected time in the four domains, scores of quality of life (Kruskal–Wallis test, years *p*=0.503, 554, 0.242, and 0.441; infected time *p*=0.059, 0.414, 0.174, and 0.367). There are also no significant differences between CD4^+^ and CD4^+^/CD8^+^ ratios in the four domains of quality of life (Mann–Whitney test CD4^+^, *p*=0.253, 0.070, 0.605, and 0.348; CD4^+^/CD8^+^ ratio *p*=0.415, 0.156, 0.263, and 0.913).

### 3.4. Quality of Life Scores in Different Constitutions

Comparisons between different constitutions in each quality of life domain score are shown in Table 4. In all domain scores of quality of life, the Excess constitution is lower than that of Special diathesis and Gentleness constitutions. (Kruskal–Wallis test: Ph, Ps, So, and *p* < 0.001).

Comparing the total and environment average score of quality of life in different constitutions, the Excess constitution is lower than the Deficiency constitution and the Deficiency constitutions are lower than the Special diathesis and Gentleness constitutions (one-way ANOVA: En, total, *p* < 0.001). The Excess constitution had the worst quality of life.

### 3.5. The OR of Abnormal CD4^+^ and CD4^+^/CD8^+^ Ratio in Different Parameters

The odds ratios (OR) of abnormal CD4^+^ and CD4^+^/CD8^+^ ratios in the parameters of years, education, occupation, constitution, and infected time are analyzed by multivariate logistic regression analysis (see Table 5). There are no significant differences between years, education level, occupation, and constitution on the abnormal CD4^+^ and CD4^+^/CD8^+^ ratio (aOR, *p* > 0.05). However, there is a significant difference in more than one year infected time compared with the ≤2 weeks, OR in abnormal CD4^+^ and CD4^+^/CD8^+^ ratio, CD4^+^ (aOR: 0.03 (95% CI, 0.01–0.09),*p* < 0.001), and CD4^+^/CD8^+^ ratio (aOR 0.07 (95% CI, 0.02–0.23, *p* < 0.001)).

### 3.6. Multiple Linear Regression of Sleep Status and Negative Mood

Multiple linear regression examined correlations between years, education, occupation, constitution, and infected time in relation to the sleep status and negative mood (Table 6).

There are no significant differences between years, education level, occupation, and infected time in the sleep status and negative mood. However, comparing Deficiency and Excess with Gentleness constitution in the sleep status and negative mood, there are significant differences.

The associations between Deficiency and Excess constitution in relation to the sleep status and negative mood are significantly different by multiple regression analysis. Sleep in Deficiency constitution (standardized regression coefficient *β* = −0.322, regression coefficient *β* = −0.701, *p*=0.001) and Excess constitution (standardized regression coefficient *β* = −0.358, regression coefficient *β* = −0.885, *p* < 0.001); negative mood in Deficiency constitution (standardized regression coefficient *β* = 0.378, regression coefficient *β* = 0.711, *p* < 0.001) and Excess constitution (standardized regression coefficient *β* = 0.482, regression coefficient *β* = 1.000, *p* < 0.001)].

Thus, sleep status in the constitutions of Deficiency and Excess is worse than in the Gentleness constitution. Negative mood in the Deficiency and Excess constitution is higher than in the Gentleness constitution, but there is no difference between special diathesis and Gentleness constitution in the sleep status and negative mood.

## 4. Discussion

### 4.1. This Study Considers HIV Infection/AIDS in MSM

According to Taiwan's CDC statistics, HIV-infected/AIDS males account for 94.79% of all cases and MSM infection rout for 65.56%, which is the largest group [24].

### 4.2. HIV-Infected/AIDS MSM Quality of Life

This study finds that the overall HIV-infected/AIDS MSM quality of life has an average score of 14.09 ± 2.37, 13.13 ± 2.83, 13.69 ± 2.94, and 14.26 ± 2.19 in the physical, psychological, social, and environmental domains. At the psychological, physical, and social level, the scores are lower than the general population in Taiwan (physical: 15.4 ± 1.81, psychological: 13.7 ± 2.07, social: 14.0 ± 2.10, and environmental: 13.1 ± 2.18) [25]. The lowest aspect of quality of life in this study is the psychological domain, followed by the social domain, consistent with previous studies on HIV-infected/AIDS persons in Taiwan: physical 13.6 ± 2.35, psychological 12.3 ± 2.63, social 12.9 ± 2.23, and environmental 13.4 ± 2.24 [4]. MSM people worry about losing their social status and feel guilty for their family, lonely, and immoral, therefore leading to lower scores in the psychosocial domain of MSM, compared to the general population. They are more likely to suffer from severe depression, anxiety, stress, low self-esteem, and social isolation [26, 27]. Therefore, HIV-infected/AIDS MSM persons need more care in the psychosocial domain.

### 4.3. Relationship between TCM Constitution and Quality of Life

In this study, Deficiency constitution accounts for a relatively large proportion, especially Yang deficiency constitution, but we found that the four quality of life domains, Excess constitution aspects (phlegm dampness, damp-heat, stagnation of qi, and blood stasis) are lower than those of the Deficiency constitution (Yang deficiency, Yin deficiency, and Qi deficiency). Previous studies have explored the relationship between the constitution and quality of life after chemotherapy of cancer and diabetes, the physical and psychological quality of life with blood stasis has also significantly deteriorated. A higher blood stasis pattern score was significantly associated with a lower PCS (physical component score) (*β* = −0.60, 95% CI: −0.73 to −0.46), lower MCS (mental component score) (*β* = −0.65, 95% CI: −0.81 to −0.49) in cancer patients [28], and lowest for the RE (role emotional) scale (F statistic = 22.94) in diabetes patients [29]. However, this study shows that the quality of life with the Excess constitution is lower than that of the Deficiency constitution. We need more research and treatment to improve the quality of life of HIV-infected\AIDS patients who are with the Excess constitution.

### 4.4. Discussion on CD4^+^, CD4^+^/CD8^+^, Sleep Status, and Negative Mood

This study also considers correlations between abnormal CD4^+^ and CD4^+^/CD8^+^ values with the patient's years, education level, occupation, TCM constitution, and infected time (see Table 5). Results show that the immune response CD4^+^ and CD4^+^/CD8^+^ ratio are related to the length of time of illness. Clearly, with the intervention of HAART drugs, the rate of recovery of CD4+ and CD4^+^/CD8^+^ ratios after illness for more than 1 year has increased, and the recovery of immunity is not closely related to the TCM constitution. This is consistent with previous studies in mainland China. Yang-deficiency constitution is related to liver toxicity, nephrotoxicity, side effects, and total mortality, but Yang deficiency is not related to CD4^+^, CD8^+^, and annual changes [30].

This study also selects the sleep status (Ph5) and negative mood (Ps6), which are the common clinical symptoms of HIV-infected/AIDS MSM, and examines their relationships with years, occupation, time of illness, and TCM constitution. Table 6 shows that although with longer HARRT treatment time, the CD4^+^ and CD4^+^/CD8^+^ ratio can be improved [31], but the sleep status (Ph5) and negative mood (Ps6) will not improve because of the longer illness. Immune recovery can improve with the intervention of HAART, but there is no improvement in the sleep status and negative mood. TCM constitution has a significant relationship with sleep status and negative mood. Therefore, the intervention of traditional Chinese medicine through the restoration of TCM constitution can reduce the incidence of negative mood, insomnia, and improve the quality of life of HIV-infected/AIDS patients.

### 4.5. Limitation

This study focuses on the relationship between constitution with immune cells and quality of life. We have additionally explored the relationship between sleep and depression with constitution in quality of life. It may be possible to further use the depression and sleep questionnaires to explore the relationship with the constitution in more detail. In our hospital, most of the patients are MSM, so the study of HIV cases is limited, if there is a chance, we can make cooperation with other hospitals to make the scope of the study wider and more comprehensive.

## 5. Conclusion

Yang deficiency constitutions were the highest ratio in this study, but the Excess constitutions Blood stasis, Qi depress, Phlegm dampness and Damp-heat are correlated with worse quality of life. Even if the immune system is restored, the psychosocial domain, sleep status, and negative mood will not improve. Therefore, in addition to antiretroviral therapy, intervention measures to improve HIV-infected/AIDS constitution through TCM may elevate the quality of life of HIV-infected/AIDS MSM patients.

## Figures and Tables

**Table 1:** Demographic distribution status of HIV/AIDS in this study.

Group (infected time)	≤2w *n* (%)	>2w–12m *n* (%)	>1y *n* (%)	Total *n*
Subjects	27 (13.3)	26 (12.8)	150 (73.9)	203
Years, mean ± Std	30.8 ± 6.1	32.0 ± 8.2	32.9 ± 6.2	32.5
Years				
20–29	11 (14.3)	13 (16.9)	53 (68.8)	77
30–39	14 (14.9)	8 (8.5)	72 (76.6)	94
40–49	2 (6.3)	5 (15.6)	25 (78.1)	32
Marital status				
Unmarried	27 (13.3)	26 (12.8)	150 (73.9)	203
Education				
Above college	18 (14.4)	16 (12.8)	91 (72.8)	125
Below high school	8 (10.7)	9 (12.0)	58 (77.3)	75
Unknown	1 (33.3)	1 (33.3)	1 (33.3)	3
Occupation				
Student	5 (14.3)	1 (2.9)	29 (82.9)	35
Employed^*∗*^	12 (12.5)	14 (14.6)	70 (72.9)	96
Unemployed^*∗∗*^	3 (11.5)	5 (19.2)	18 (69.2)	26
Others	7 (15.2)	6 (13.0)	33 (71.7)	46
CD4^+^				
<292	18 (47.4)	9 (23.7)	11 (28.9)	38
≥292	9 (5.5)	17 (10.3)	139 (84.2)	165
CD4^+^/CD8^+^ ratio				
<0.6	23 (25.3)	20 (22.0)	48 (52.7)	91
≥0.6	4 (3.6)	6 (5.4)	102 (91.1)	112

^*∗*^Service industry, business, public employee, worker and ^*∗∗*^unemployed and free industry.

**Table 2:** Different age, rank, infected time, CD4^+^, and CD4^+^/CD8^+^ ratios for different constitution distributions.

	Deficiency type *n* (%)	Excess type *n* (%)	Special diathesis types *n*(%)	Gentleness type *n* (%)	Total *n*	*p* value
Subjects	82 (40.4)	56 (27.6)	21 (10.3)	44 (21.7)	203	
Years^†^						
20–29	29 (37.7)	19 (24.7)	12 (15.6)	17 (22.1)	77	0.63^†^
30–39	39 (41.5)	29 (30.9)	6 (6.4)	20 (21.3)	94	
40–49	14 (43.8)	8 (25.0)	3 (9.4)	7 (21.9)	32	
Infected time^†^						
≤2w	10 (37.0)	4 (14.8)	3 (11.1)	10 (37.0)	27	0.18^†^
>2w–12m	14 (53.8)	7 (26.9)	3 (11.5)	2 (7.7)	26	
>1y	58 (38.7)	45 (30.0)	15 (10.0)	32 (21.3)	150	
CD4^+‡^						
<292	13 (34.2)	11 (28.9)	3 (7.9)	11 (28.9)	38	0.62^†^
≥292	69 (41.8)	45 (27.3)	18 (10.9)	33 (20.0)	165	
CD4^+^/CD8^+^ ratio^‡^						
<0.6	37 (40.7)	25 (27.5)	12 (13.2)	17 (18.7)	91	0.58^‡^
≥0.6	45 (40.2)	31 (27.7)	9 (8.0)	27 (24.1)	112	

^†^Fisher's exact test, ^‡^chi-square test, deficiency type (Yang deficiency, Yin deficiency, and Qi deficiency), and excess type (phlegm-wetness. Wh: wetness-heat, Bs: blood stasis, Sd: special diathesis, and G: gentleness type).

**Table 3:** Quality of life scores by age, rank, infected time, CD4^+^, and CD4^+^/CD8^+^ ratio.

	Subjects	Physical		Psychological		Social relationship (TW)		Environment (TW)	
		Median (IOR)	*p* value	Median (IQR)	*p* value	Median (IQR)	*p* value	Median (IQR)	*p* value
Average scores (mean ± SD)	203	14.09 ± 2.37		13.13 ± 2.83		13.66 ± 2.78		14.26 ± 2.19	
Years^†^									
20–29	77	14.3 (12.6–16.0)	0.503	13.3 (11.3–16.0)	0.554	14.0 (12.0–16.0)	0.242	14.7 (12.9–15.6)	0.441
30–39	94	14.3 (12.6–15.4)		13.3 (11.3–14.7)		13.0 (12.0–15.0)		14.2 (12.9–15.6)	
40–49	32	14.9 (12.6–15.4)		13.3 (12.0–14.7)		14.0 (12.0–15.0)		14.7 (13.6–16.0)	
Infected time^†^									
≤2w	27	14.3 (12.0–16.0)	0.059	13.3 (10.7–16.0)	0.414	15.0 (13.0–16.0)	0.174	15.1 (13.8–16.4)	0.367
>W∼12m	26	13.1 (11.4–14.9)		12.3 (10.7–14.7)		13.0 (12.0–15.0)		14.4 (13.3–15.1)	
>1y	150	14.3 (12.6–16.0)		13.3 (11.3–15.3)		14.0 (12.0–15.0)		14.2 (12.4–15.6)	
CD4+^‡^									
<292	38	13.7 (12.0–15.4)	0.253	12.0 (10.7–14.7)	0.070	14.0 (12.0–16.0)	0.605	14.0 (12.9–15.6)	0.348
≥292	165	14.3 (12.6–16.0)		13.3 (11.3–15.3)		14.0 (12.0–15.0)		14.7 (12.9–15.6)	
CD4+/CD8+ ratio^‡^									
<0.6	91	14.3 (12.0–16.0)	0.415	12.7 (11.3–15.3)	0.156	13.0 (12.0–15.0)	0.263	14.7 (12.9–15.6)	0.913
≥0.6	112	14.3 (12.6–16.0)		13.3 (12.0–15.3)		14.0 (12.0–16.0)		14.4 (12.9–15.6)	

^†^Kruskal–Wallis test and ^‡^Mann–Whitney *U* test.

**Table 4:** Quality of life scores in 9 TCM constitution distributions.

Subjects	Deficiency	Excess	Special diathesis	Gentleness	Total	Post hoc
	82	56	21	44	203	
	Mean ± SD	Mean ± SD	Mean ± SD	Mean ± SD	*p* value	
Physical^a^	13.74 ± 2.09	**12.88** **±** **2.59**	15.05 ± 1.23	15.82 ± 1.78	<0.0001	2 < 3, 4; 1 < 4
Psychological^a^	12.84 ± 2.52	**11.54** **±** **2.76**	14.06 ± 2.51	15.27 ± 2.07	<0.0001	2 < 3, 4; 1 < 4
Social relationship (TW)^a^	13.32 ± 2.73	**12.57** **±** **2.76**	15.05 ± 2.16	15.02 ± 2.41	<0.0001	2 < 3, 4; 1 < 4
Environment (TW)^b^	14.16 ± 2.15	**13.29** **±** **2.17**	15.09 ± 1.27	15.28 ± 2.10	<0.0001	2 < 3, 4; 1 < 4
Total^a^	54.05 ± 8.11	**50.28** **±** **8.65**	59.25 ± 5.99	61.40 ± 7.49	<0.0001	2 < 1 < 3, 4

^a^Kruskal–Wallis test unusual distribution, without mother number. ^b^One way ANOVA: normal distribution deficiency constitution: Yang deficiency, Yin deficiency, and Qi deficiency. Excess constitution: phlegm-wetness, wetness-heat, and blood stasis constitution; Sd: special diathesis constitution; G: gentleness constitution.

**Table 5:** Multivariate logistic regression analysis of the risk factor for abnormal CD4+ and CD4+/CD8+ ratio.

Independent variables	CD4^+^		CD4^+^/CD8^+^ ratio	
	Adjusted OR (95% CI)	*p* value	Adjusted OR (95% CI)	*p* value
Years				
20–29	-	-	-	-
30–39	2.09 (0.75–5.84)	0.158	1.48 (0.72–3.04)	0.288
40–49	0.37 (0.06–2.28)	0.282	1.06 (0.39–2.89)	0.904
Education				
Above college	-	-	-	-
Below high school	0.88 (0.33–2.36)	0.797	0.86 (0.44–1.68)	0.656
Occupation				
Student	-	-	-	-
Employee	0.41 (0.06–2.68)	0.349	0.75 (0.23–2.41)	0.625
Unemployed	1.34 (0.35–5.19)	0.674	0.57 (0.23–1.42)	0.224
Others	0.45 (0.09–2.29)	0.334	0.51 (0.18–1.47)	0.214
Constitutions				
Gentleness	-	-	-	-
Deficiency	0.47 (0.14–1.63)	0.232	1.37 (0.56–3.34)	0.492
Excess	1.23 (0.35–4.32)	0.753	1.61 (0.63–4.12)	0.325
Special diathesis	0.47 (0.08–2.81)	0.411	2.58 (0.74–9.05)	0.138
Infected time				
≤2w	-	-	-	-
>2w∼12m	0.26 (0.07–0.99)	0.047	0.57 (0.13–2.44)	0.447
>1y	0.03 (0.01–0.09)	**<0.001**	0.07 (0.02–0.23)	**<0.001**

OR: odds ratio.

**Table 6:** Multiple regression of the risk factor for sleep status and negative mood.

Independent variables	Sleep			Negative mood		
	*β*	Standardized beta	*p*	*β*	Standardized beta	*p*
Years						
20–29	-	-	-	-	-	-
30–39	−0.146	−0.068	0.381	0.005	0.002	0.974
40–49	0.073	0.025	0.747	0.020	0.008	0.918
Education						
Above college	-	-	-	-	-	-
Below high school	0.181	0.082	0.245	0.006	0.0037	0.962
Occupation						
Student	-	-	-	-	-	-
Employee	0.156	0.048	0.574	−0.132	−0.047	0.577
Unemployed	0.214	0.100	0.326	−0.200	−1088	0.283
Others	0.268	0.105	0.278	−0.012	−0.005	0.956
Constitutions						
Gentleness	-	-	-	-	-	-
Deficiency	**−0.701**	**−0.322**	**0.001**	**0.711**	**0.378**	**<0.001**
Excess	**−0.855**	**−0.358**	**<0.001**	**1.000**	**0.482**	**<0.001**
Special diathesis	−0.117	−0.033	0.680	0.485	0.157	0.047
Infected time						
≤2w	-	-	-	-	-	-
>2w∼12m	−0.255	−0.079	0.389	0.083	0.030	0.744
>1y	0.193	−0.079	0.384	−0.037	−0.018	0.845

## Data Availability

If you need our original data, you can directly contact the first author, the e-mail address is: DAI55@tpech.gov.tw.
